# Supplementary material for: Comparative Characterization of Different Molecular Formats of Bispecific Antibodies Targeting EGFR and PD-L1
Source: Pharmaceutics. 2022 Jun 29;14(7):1381. doi: 10.3390/pharmaceutics14071381 (PMC9325241; doi:10.3390/pharmaceutics14071381)

Figure S1. Unprocessed western blot image for Figure 2D.

MDA-MB-231

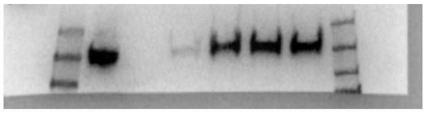

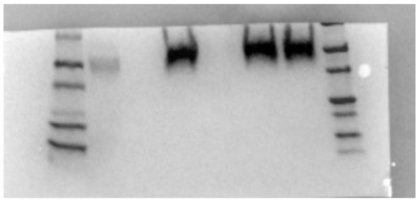

BT-20

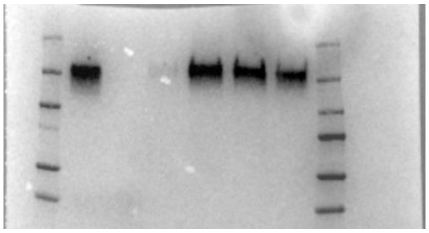

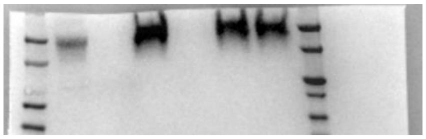

Figure S2. Unprocessed western blot image for Figure 8A, 8B, 8C

Figure 8A unprocessed western blot images

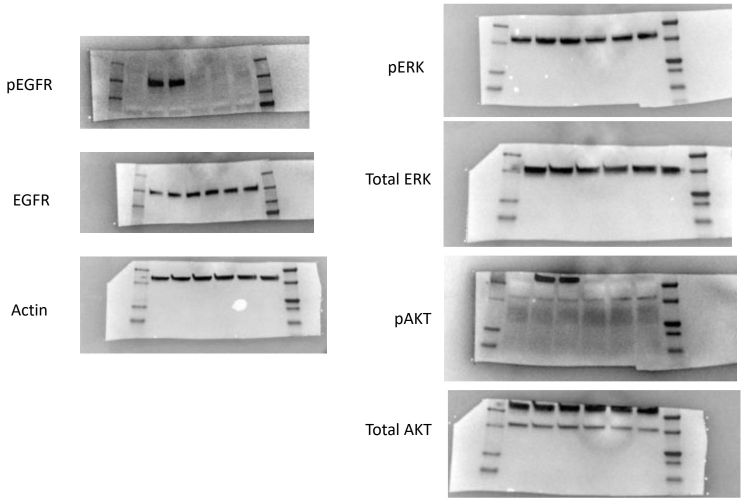

Figure 8B unprocessed western blot images

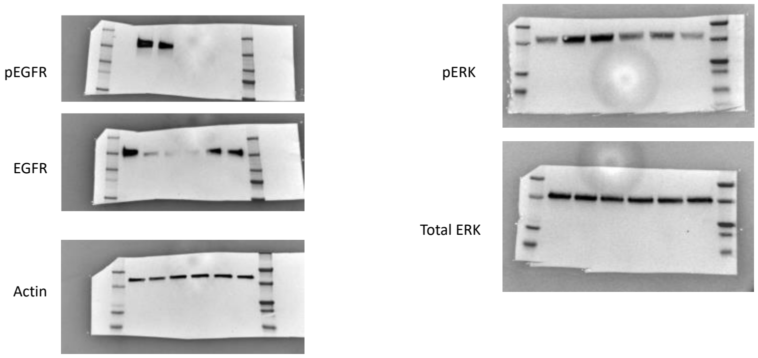

Figure 8C unprocessed western blot images

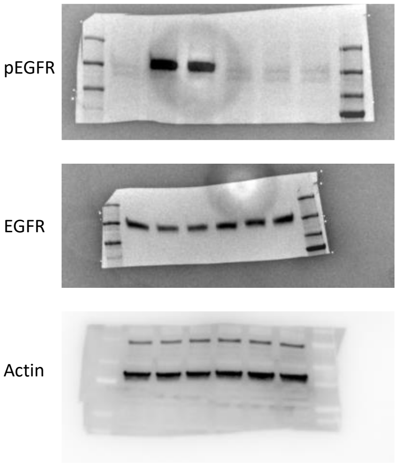

Supplement: Supplementary file 1 [file pharmaceutics-14-01381-s001.zip › pharmaceutics-1747822-supplementary.pdf]
